# How Do Master Weightlifters Train? A Transnational Study of Weightlifting Training Practices and Concurrent Training

**DOI:** 10.3390/ijerph19052708

**Published:** 2022-02-25

**Authors:** Marianne Huebner, Friedrich Faber, Katharine Currie, Thomas Rieger

**Affiliations:** 1 Department of Statistics and Probability, Michigan State University, East Lansing, MI 48824, USA; 2 Bundesverband Deutscher Gewichtheber, Masterausschuss Gewichtheben, 69181 Leimen, Germany; faber.friedrich@t-online.de; 3 Department of Kinesiology, Michigan State University, East Lansing, MI 48824, USA; curriek4@msu.edu; 4 Faculty of Business and Sport, University of Europe for Applied Sciences, 58638 Iserlohn, Germany; thomas.rieger@ue-germany.com

**Keywords:** weightlifting, sport, older adults, aging, sex differences, concurrent training, geographic differences

## Abstract

(1) Background: The wide range of preparedness, physical fitness, and capabilities of older athletes makes it challenging to recommend general training programs for this cohort ranging from ages 35 to 80 and older. Weightlifting has enjoyed an unprecedented growth in recent years, especially among women. The objectives of this study are to describe age and sex differences in self-reported training regimens and concurrent training for Masters weightlifters and investigate regional differences. (2) Methods: A total of 1051 Masters weightlifters from Australia, Canada, Europe, and the USA completed an online survey that included questions on sport history and training practices. (3) Results: A training session typically lasted 1.5 to 2 h for both sexes across all ages. Weightlifters engaged in concurrent training (66.9%), especially in endurance training (24.9%) and CrossFit (36.4%), but the proportions differed across geographic regions. Older females maintained training 4 days per week, while older males reduced this to 3 days per week. (4) Conclusions: Weightlifting training practice of Masters athletes was remarkably consistent across ages, but concurrent training differed between males and females and across regions. This study provides helpful information for athletes, coaches, and sport organizations about the variation in weightlifting training practices and concurrent training of older athletes.

## 1. Introduction

Weightlifting is a competitive sport consisting of the performance of snatch and clean and jerk in competitions governed by rules by the International Weightlifting Federation (IWF). Similar to the popularity of resistance training as a physical activity, the sport of weightlifting has enjoyed an unprecedented increase in participation in recent years, especially among women [1]. For example, from 2015 to 2019 the participation rate in the USA National Masters Weightlifting Championships has increased dramatically, from 244 to 718 competitors [2]. Moreover, there were 196 weightlifters who competed in the German National Masters Championships 2021, the highest number in recent years. Older athletes are making use of increased opportunities to participate in competitive sports. This is despite a greater risk of chronic diseases with older age that need to be considered when designing training regimens [3].

The wide range of preparedness, physical fitness, and capabilities of Masters weightlifters, aged 35 years and older, makes it challenging to recommend general training practices. Older athletes may have less access to qualified coaches or experience ageism in the fitness industry [4]. Prior literature has focused on performance or training for younger elite athletes [5,6,7,8,9], or on performance decline with aging [10,11]. Weightlifting training programs include appropriate choices of exercises to develop technical skills and explosive strength in addition to competition-specific exercises integrated with recovery phases and proper nutrition. A weightlifting training session consists of competitive lifts, such as snatch and clean and jerk, and accessory lifts such as hang snatch or clean from blocks, among others [12,13]. These are typically followed by strength exercises such as squats or presses. Supplementary exercises may be used that can include pull-ups, core exercises, or exercises at weight machines. General concepts of weightlifting program designs over a time period include specificity of exercises, overload, and variability, but program designs vary among internationally competitive weightlifters [9,12,13,14]. Older athletes may respond more slowly to training stimuli than younger athletes and take longer for recovery and concurrent training may be needed [15]. Training for Masters athletes varies widely because of demands on time, health reasons, or physical and functional capacity.

To date, in Masters athletes little is known about training practices, frequency, length, composition of training sessions, and whether concurrent training with aerobic exercises or cross-training in other sports is undertaken. There are many ways to design successful training programs. Different volumes, intensities, frequencies, or selection of exercises are used in training programs by successful Master weightlifters, who have found ways to experiment, modify, and adapt to their needs [16]. Preconceived notions of what activities and training volumes older athletes can tolerate may be discussed on social media and in blog posts. However, coach education programs include only limited information about the needs of older athletes [17]. Thus, coaches and Masters weightlifters are often left to their own devices to choose training programs and gather information from different sources that may not always be appropriate for older athletes. Some weightlifters follow their own programs, modify available subscription programs, or make use of remote coaching. While cross-training has been studied for endurance athletes [18,19], little is known about cross-training for weightlifters, particularly for those in older age groups. Weightlifting athletes compete in body-weight categories and thus are often careful about diet before competitions. Little is known about how much importance weightlifters assign to nutrition for their training. To answer some of these questions on how Masters weightlifters train and their perception of nutrition, we surveyed Masters weightlifters from six countries. Awareness of the variations in current training practices of older athletes would help coaches to promote positive habits and prevent overtraining and injuries. Describing their self-reported training practices is an important first step in understanding how Masters weightlifters should train to optimize their performance and health.

The aim of this study was to describe self-reported training practices of Masters weightlifters, including the proportion of time spent engaging in concurrent training with aerobic exercises, or cross-training in other sports. A secondary aim was to explore regional differences in training practices.

## 2. Materials and Methods

### 2.1. Sample

Participants were weightlifters in the Masters category who turned 35 years of age or older during 2021 in Australia (AUS), Canada (CAN), Germany (GER), Great Britain (GBR), Spain (ESP), and the United States (USA). Individuals were invited to participate in the study through emails and newsletters by the national governing bodies of weightlifting, and via online platforms including Facebook and Instagram. The survey was administered online via Qualtrics (Provo, UT, USA). The study protocol was approved by the Michigan State University Human Research Ethics Committee and all participants provided online informed consent. Of the 1120 respondents, 65 were excluded from the study because of incomplete responses and four study participants were excluded because of indicating a sex other than male or female. This population was previous described in [20].

### 2.2. Measures

Information about sport participation and physical activities prior to starting weightlifting was collected, as well as whether the weightlifters currently engaged in these activities in addition to their current weightlifting training. Resistance training, power lifting, ball sports, endurance, fitness, mobility (e.g., yoga or Pilates), and martial arts were included as options, as well as an open-ended question asking about other activities. Questions about weightlifting training were of the type, “On average how much time in your typical training session was devoted to…?” and asked about elements of weightlifting training warm-up, (snatch, clean and jerk, and accessories such as hang snatch or clean from blocks), strength exercises (squats, presses), additional exercises (pull-ups, core, machines, etc.), and cool-down with options 0–15 min, 15–30 min, 30–45 min, 45–60 min, or more than 60 min. A Likert scale (strongly agree, agree, neither agree nor disagree, disagree, strongly disagree) was used to ask participants whether following a training-specific nutrition program was important for their weightlifting training (i.e., recovery, muscle increase).

### 2.3. Statistical Analyses

Continuous variables were summarized with median and quartiles, and categorical variables were summarized with frequencies and percentages, stratified by age groups and sex. Age groups were defined as 35 to 44 years, 45 to 59 years, and 60 years and older. The age groupings were chosen to align with Master age categories that are in 5-year increments but grouped so as to separate the younger age group with the largest recent increase in participation, and to study a combined older age group covering a wide age range because of the relative paucity of data for older ages. Pearson’s chi-square test was used to compare distributions of categorical variables, and the Wilcoxon rank sum test was used to compare distributions of numerical variables. K-means clustering was used to explore possible subgroups based on sex, age, training variables, and participation in other sports. The number of optimal clusters was determined with a silhouette plot. The silhouette value is a measure of how similar an object is to its own cluster compared to other clusters [21]. Statistical analyses were performed utilizing the statistical software R v. 4.0.3 [22] and the package *factoextra* v.1.0.7 [23]. For all analyses, a *p*-value of 0.05 was considered statistically significant.

## 3. Results

Table 1 lists the demographics by age and sex of 1051 participants from Australia (8.6%), Canada (11.8%), Europe (inclusive of GER, GBR and ESP; 17.8%), and the USA (61.8%). Overall, 523 females participated in the survey (49.8%). There were more than 50% females in all regions, except in Europe, where females accounted for only 31% of the participants. In the oldest age group, males started weightlifting training at a median age of 19 years (quartiles: 14, 55), while females started weightlifting training at age 55 years (quartiles: 49, 60). In the younger age group, the median starting age was comparable between males and females at 32 and 34 years, respectively. Over 90% of the participants have competed in weightlifting competitions. There were more male coaches than female coaches (27.4% versus 20.3%, (χ^2^ = 11.5, df = 4, *p* = 0.008)); at ages 60 years and older this included 26.1% male and 15.1% female coaches. The proportions of referees were 20.8% at ages 35–44 years, 26.5% at 45–59, and 37.0% at 60+ years, respectively, thus higher in the oldest age group (χ^2^ = 29.9, df = 6, *p* < 0.001). There are fewer female referees in Europe (25.6% male versus 8.6% female referees, respectively, *p* = 0.041), while in the USA the proportions for male and female referees were similar (31.0% versus 27.4%, respectively, (*p* = 0.361). The majority live close (less than 30 min) to their main training location (64–74%) or train at home. Males were more likely to train at home compared to females, in particular in the oldest age group (29.3%).

**Figure 1:**
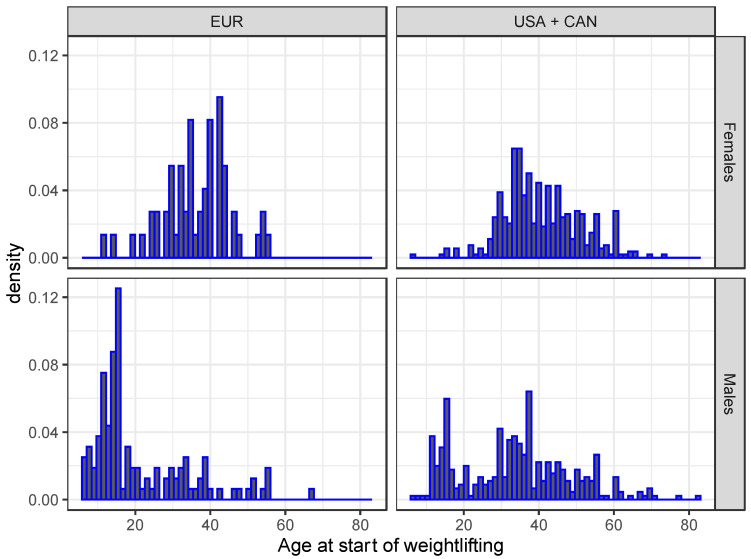
Distribution of age at start of weightlifting in North America (USA + CAN) and Europe (EUR).

The median age (quartiles) at the start of weightlifting for females was 39 years (34, 47 years) and for males 32 years (16, 40 years) (*p* < 0.001). There was a distinct subgroup of males who started weightlifting before age 20 years both in North America (24.5%) and, in a larger proportion, in Europe (64.5%) (Figure 1). There were no females who started weightlifting in Europe at age 60 years or older, but 5.5% of the females accounted for these older beginners in the USA and Canada. The corresponding proportions for males were 0.8% and 5.1%, respectively.

### 3.1. Training Habits, Frequency, and Length

Training location, frequency and time are presented in Table 2. Males and females of all ages primarily trained at weightlifting clubs (54–64%), although a combination of locations was utilized by many. CrossFit boxes were a typical training location, especially for females across ages or for males in the 35–44-year-old age group. Of the females in the oldest age group, 40% reported typically training in CrossFit boxes. Training at home was undertaken by 20–40% of the participants. Males, regardless of age, trained more often at home than females. Training at fitness centers was less common, regardless of age and sex. Female weightlifters were guided by training programs from coaches, while males, with increasing age, designed their own programming, especially in the oldest age group (68%). The number of training days per week dedicated to weightlifting was typically 3 to 4 days per week. Older males reported training 3 days per week; younger athletes, especially females, reported training 5 days per week. The duration of the training, regardless of age and sex, was preferably 1.5 to 2 h per session, although 1 to 1.5 h becomes more common with increasing age.

**Figure 2:**
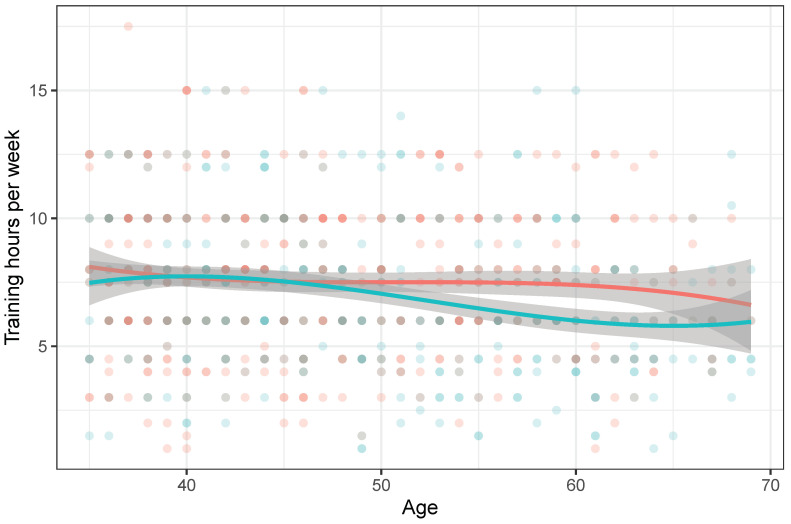
Training hours per week by age and sex (females—red, males—green).

Training hours per week were defined by the maximum of the categorical intervals of training sessions (1, 1.5, 2, 2.5 h) multiplied by the number of training days per week. Weightlifting training lasted on average 7.5 h per week for ages 35–44 and 45–59, and 6 h per week for ages 60 years and older. This estimate does not distinguish between international-level athletes and those who only compete in local competitions. Cubic splines with confidence intervals (grey shaded areas) were fitted to examine the association of age with training hours per week (Figure 2). This illustrates that the weekly training hours decrease for males with age, while it stays the same for females; females older than 50 years may train more than males or possibly tolerate more. It is unclear what the average training hours may be for females older than 65 years because of the sparsity of female participation in this cohort.

The smoothed lines show the trend across age with grey-shaded confidence intervals. Circles represent training hours of individuals (green for males and red for females).

A weightlifting training program consists of warm-up, competition lifts, strength exercises, possible supplementary exercises, and cool-down. A warm-up time of 0–15 min was commonly reported. Competition-related lifts were typically performed for 30–60 min. Time for strength exercises varied between 15 and 45 min, regardless of age and sex. Additional exercises took about 15–30 min, and 0–15 min at older ages. Over 90% of the participants reported the cool-down interval to be 0–15 min, irrespective of age and sex.

Over 50% of the females strongly agreed that training-specific nutrition was important for their weightlifting training (muscle increase or recovery), while fewer males (e.g., 38% of males 60 and older) rated this as “strongly agree”. Consistent across ages, 26.0% of the females relied on a nutrition coach compared to 9.4% of the males. Overall, 20.3% obtained nutrition advice from coaches, teammates, or others.

Regional differences are presented in Table 3. Compared to other regions, in Europe fewer weightlifters trained at home (10.3% of the females and 18.3% of the males) and most often in weightlifting clubs (75.9% of the females and 80.2% of the males). Over 40% of the males in Australia, Canada, and the USA reported that they trained at home, but multiple locations were possible. The largest proportion of males utilizing their own training program were Europeans (71.4%). In Canada and Europe, 3 training days per week was more common than in Australia and the USA where 4 days prevailed. There were fewer training hours per week in Canada and Europe (median 6 h) than in Australia and the USA (median 7.5 h). A training session typically lasted 1.5 to 2 h across regions and sex. However, 28.0% of the males in Europe reported their training session to be more than 2 h. Overall, the time spent on competition lifts and strength exercises was similar across regions. Competition lifts in a training session typically lasted between 30 and 60 min, although more than 20% of males and females trained more than 1 h. While females agreed or strongly agreed about the relevance of nutrition for training (85.3%), fewer males found nutrition relevant (74.8%), and this differed across regions: 86.3% in Australia, 83.8% in the USA, 64.8% in Canada, and 54.0% in Europe.

### 3.2. Sport Participation Prior to Weightlifting

To the question, “Have you participated in sports or physical activities before you started weightlifting?” more than 90% of the survey participants reported that they had participated in other sports before starting weightlifting (Table 4). CrossFit was most common in the youngest age group, especially among females (75.0% females and 63.2% males). Other popular activities were ball sports, such as soccer or volleyball, and bodybuilding/strength training (37%). In the age group 45–59 years, CrossFit was also most common for females (69.8% for females and 42.5% for males), while many males had prior experience with ball sports (53.5%) and had already engaged in strength training (41.0%). In the 60+ years group, CrossFit, endurance, and fitness were among the most common sports for females prior to weightlifting while ball sports and strength training was reported by males. More females than males practiced mobility activities such as yoga and Pilates across all age groups.

There were regional differences. The largest proportion of weightlifters who were not engaged in a sport prior to weightlifting were from Europe (13.6%), while this proportion ranged from 6 to 10% in other countries (χ^2^ = 11.8, df = 3, *p* = 0.007). CrossFit was commonly reported in Australia (49.5%), Canada (50.8%), and the USA (64.9%), while this accounted for only 22.4% of the European respondents (χ^2^ = 108.5, df = 3, *p* <0.001). Ball sports and strength training were also common in the different countries.

### 3.3. Sport Participation in Addition to Weightlifting

To the question, “In a typical week have you also participated in other physical activities/sport in addition to weightlifting before the pandemic?” 66.8% of the participants reported additional sport activities concurrent to weightlifting (Table 5). CrossFit remained the most common sport for females (47.8% for ages 35–44 years and 46.4% for ages 45–59 years). Endurance exercises, such as cycling, swimming, running, and walking, were popular as additional physical activities reported by over 20% of the weightlifters. This was similar across age, sex, or region. CrossFit and endurance were equally represented among 60+ year old females with 31.5% and 30.1%, respectively.

Masters athletes in different geographic regions engaged in sport or physical exercises in addition to weightlifting, 68.1% in Australia, 72.6% in Canada, 55.7% in Europe, and 68.6% in the USA, (χ^2^ = 13.2, df = 3, *p* = 0.004). CrossFit was popular in the USA (40.1%), Canada (40.3%), and Australia (37.4%), but less so in Europe (20.3%) (χ^2^ = 25.9, df = 3, *p* < 0.001). Additional strength training, including powerlifting, was more common in Australia and Canada than in Europe or the USA (χ^2^ = 15.0, df = 3, *p* = 0.002).

### 3.4. Characteristics of Subgroups of Weightlifters

To explore whether there were subgroups of weightlifters with specific training regimens we used a K-means clustering algorithm with variables age, sex, sport participation, and training-related variables. This identified two subgroups (average silhouette width 0.25) with the explained variation for two dimensions 22.3%. The characteristics present in cluster 1 (*n* = 784) were female (63.8%), younger (mean age 47), participated in CrossFit, and engaged more in supplementary exercises and mobility exercises compared to individuals in cluster 2 (*n* = 237), who were largely male (94.3%) and older (mean age 62).

## 4. Discussion

Masters weightlifters (aged 35 years and older) from six countries were surveyed about how they train and their perception of nutrition in relation to their training. Females accounted for more than 50% of the participants in all regions except Europe (31%). The aim was to investigate self-reported practices for the training structure for Masters weightlifters, including the use of concurrent training with aerobic exercises or cross-training in other sports. Such information might help athletes, coaches, and medical professionals to understand the possibilities and limitations of program designs for the aging athlete to optimize performance, fitness, and health, taking strength and aerobic qualities into account. Since coaching Masters athletes requires knowledge of training capacity and variability and the unique needs of older athletes [17] (“Coaching Master Athletes” and references therein), and little is known about the training regimens of older weightlifting athletes, describing their self-reported training practices is an important first step in understanding how Masters weightlifters should train to optimize their performance and health.

There are regional differences in sport participation or engagement in vigorous physical activities that can in part be explained by cultural norms, socioeconomic status, or availability of facilities. The World Masters Weightlifting Championship was held virtually in 2021 and thus barriers caused by travel and cost were lowered. As a result, this event included athletes from 102 countries, almost double the number of countries represented in prior years, and the proportion of female weightlifters was 47%, with large differences between countries. Among the six participating countries in this survey, the proportion of females was 12% for Germany, 30% for Spain, 67% for the UK, 58% for Australia, 60% for the USA, and 63% for Canada.

### 4.1. Weightlifting Training

Weightlifting is a high-intensity competitive sport, requiring speed, explosive power, and technical skills that result in physiological adaptations of the musculoskeletal and cardiovascular systems [9,24]. There are numerous training programs and practices among internationally competitive weightlifters with younger athletes performing resistance exercise of the same major muscle groups 6–7 days per week [9]. General concepts of weightlifting training programs include specificity of exercises, overload, and variability [9,12,13]. Peak performance in weightlifting is reached in their mid- to late twenties [8,25] and declines over time. This decline differs between sports depending on whether the emphasis is on endurance, strength, or anaerobic function, and hormonal changes in the transition to menopause account for some sex differences [10,11]. While the normal physiology of aging is likely the primary factor driving the performance decline, changes in training volume and intensity may also be contributing factors. Master athletes have competing commitments because of family and work-related responsibilities, so they may have limited time available to devote to training [26].

In this study, the majority of the time in weightlifting training sessions was spent on competition lifts and derivatives (35%), which comprise snatch, clean and jerk, hang snatch, clean from blocks, etc., followed by strength exercises (26%). Additional training time was spent on supplementary exercises that targeted synergistic muscle groups, and short warm-up and cool-down periods. A typical session lasted 1.5 to 2 h across age and sex, and the training frequency was 3 or 4 days per week, although 21% trained for 5 days and 6% for 6 or 7 days. Overall, females were more likely to train 4 days per week across all ages, while males reduced their training to 3 days per week at older ages. Weightlifting training lasted on average 6–10 h per week for age 35–59 years and 4–7 h per week for ages 60 years and older, which is similar to runners and swimmers in their mid-50s who average about 6–10 h weekly [27]. There were regional differences, for example, Europeans trained fewer days per week, but the sessions were longer for European males. This training volume did not account for additional physical activities. However, our analysis revealed two clusters of Masters weightlifters that differed in their training habits. The characteristics of one group was a younger age (mean age 47 years) who engaged in more supplementary and mobility exercises and CrossFit, while the other group consisted largely of older males (mean age 62 years) who focused more on the classic lifts.

### 4.2. Concurrent Training and Cross-Training

The majority of weightlifters in this survey (64%) were involved in concurrent training defined as the combination of strength and aerobic or endurance training or in cross-training that includes training in sports other than weightlifting. There were regional differences, with fewer Europeans (55%) undertaking physical activities in addition to their weightlifting training. The type of endurance training or sport that weightlifters choose to engage in may be based on sport history or availability, or to mitigate shortcomings of the primary sport. Endurance exercises, such as cycling, swimming, running, and walking, were reported by over 24% of the weightlifters, while fitness activities in general were reported by 20% of the respondents. This was similar across ages and sex. While we did not investigate the number of days spent on concurrent or cross-training, in a prior survey of USA Masters weightlifters in January 2020 [2], walking was the most frequent physical activity with a median of 4 days per week (quartiles: 2, 7 days), followed by CrossFit (median 2 days per week; quartiles: 1, 4 days), endurance activities (median 2 days; quartiles: 2, 4 days), and mobility activities such as yoga/Pilates (median 2 days; quartiles: 1, 3 days). Ball sports were practiced one day (quartiles: 1, 3 days) during a typical week.

Both endurance/aerobic and strength training are recommended for fitness and physical health (WHO 2020) [28], with the combination of both training modalities having greater health benefits than either of these modalities alone in older adults [29,30]. While many athletes and leisure exercisers engage in both, the compatibility of the endurance and strength continues to be of interest because of the potential effects of endurance exercise on improving strength [31].

Moderators of training adaptations and possible interference effects of concurrent training have been discussed, such as the timing of and intervals between these training modalities, frequency, and type of aerobic exercises (e.g., running, swimming, cycling), experienced in the sport, training status, and older age. However, maximal strength has been shown to be adversely affected by concurrent training depending on training frequency or duration [32]; high volume and moderate-intensity endurance training may negatively affect training adaptations induced by strength training while low-volume, short bouts may have lower or no effect on resistance-training-induced adaptations [31,33]. Trained individuals may also experience more negative effects on strength gains with concurrent training compared to untrained individuals [34]. However, data presented in a recent literature review on concurrent endurance and strength training suggest this approach to training does not compromise muscle hypertrophy and maximal strength development irrespective of frequency, age (less than 40 years versus older), and untrained or trained status [35]. The conflicting messaging about the interfering effects of concurrent training is likely attributed to differences in the experimental settings and study participants. Both endurance and strength exercises can be incorporated in training programs with a low risk of interference effects if one considers volume, intensity, and frequency of each exercise [35,36], but for competitive weightlifters, it is important to consider that concurrent training may compromise explosive power as measured by jump height and other rapid force production, and thus they may benefit from separating endurance and strength training to minimize the potential interference effects [35,37,38].

It is unclear whether there are sex differences in interference effects with concurrent training. Male athletes have more muscle mass and less body fat than female athletes. The magnitude of sex differences in weightlifting performances range from 25–30% depending on body mass for adults in their 20s but can be higher for Masters weightlifters [2]. However, women have been shown to be more resistant to muscle fatigue and thus interference effects from concurrent training may be smaller for women [39].

Over 90% of the participants reported being active in sport or physical activities prior to weightlifting. CrossFit provided an entry point for females into weightlifting, while males had more experience with strength exercises and team sports prior to starting weightlifting. CrossFit continued to be practiced in addition to weightlifting by more than 40% of the females (31% over age 60), while this is the case for less than 30% of the males (13% over age 60). Ball sports in addition to weightlifting were practiced by 13% of the males, but only 5% of the females. Of those who participated in ball sports, 91% had already done so prior to weightlifting. Like concurrent training, CrossFit includes both endurance and strength activities, and therefore athletes should consider the potential interfering effects on their weightlifting training program.

While the quantification of training volume is important to understanding performance, it may also be useful in understanding health-related metrics in this unique population. As previously described, older adults are encouraged to engage in aerobic/endurance activities, strength-based activities, and functional balance activities [28], as these modes of training have been shown to promote healthy aging and well-being and reduce the risk of chronic diseases [40]. For example, studies have shown improved measures of cardiovascular health [41], such as arterial compliance [41,42], endothelial function [41], and left ventricular structure and function [43], in older active adults compared to their sedentary peers. However, higher volumes of physical activity or exercise beyond what is recommended do not necessarily infer greater benefits. In particular, the “Extreme Exercise Hypothesis” describes a U or reverse J-shaped relationship between physical activity volumes and cardiovascular health outcomes with higher volumes associated with increased coronary artery calcification, myocardial fibrosis, and atrial fibrillation, to name a few [44]. It is important to note that much of the literature is based on excessive endurance exercise, not resistance or concurrent exercise. The WHO guidelines recommend a minimum of 150–300 min of moderate-intensity aerobic physical activities per week and strength training for major muscle groups two times per week for ages 18-64 years and, in addition, incorporate multicomponent physical activity with an emphasis on functional balance and strength training 3 or more days per week for ages 65 and older [28]. Based on the current survey responses, respondents appear to be exceeding the strength-based physical activity WHO guidelines, given the majority trained 6–10 h per week. While most respondents also identified they were concurrently training or engaging in CrossFit (64%), it is difficult to characterize these aerobic/endurance activities as meeting or exceeding physical activity guidelines given that we did not ask how many hours per week or at what intensity these activities were undertaken. Weightlifting has been associated with excellent balance performance compared to aerobic activities (i.e., running) [45] and improved range of motion [46,47], and thus it is likely that the functional balance recommendations are being met with weightlifting training. In addition, activities including yoga and Pilates were reported by 19% of the female and 5% of the male participants. In the end, quantifying training volumes in Masters athletes engaging in weightlifting with or without concurrent or CrossFit training is important to fully understanding the potential effects on health-related outcomes.

### 4.3. Nutrition

Weightlifting is a sport with body-weight categories in competitions and, therefore, many athletes are purposefully approaching their diets with a goal of losing or gaining weight before competitions. Weightlifting training comes with a high metabolic cost and athletes recognize that their protein needs are higher than those of the general population, but there may be gaps in their sports nutrition knowledge [48]. Dietary protein intake and optimizing timing of protein intake has received considerable attention [49]. While weightlifters often meet the protein and fat requirements, there may be a macronutrient imbalance that could result in suboptimal training gains [9]. Since aging increases general inflammation and chronic diseases, the topic of exercise and nutrient interaction becomes important for Masters athletes. Adequate protein and energy intake and higher demand for specific nutrients may affect nutrient absorption, training capacity, and bone and muscle mass, and reduce inflammatory burden in older athletes [50]. Females have different nutritional needs than men because of different body composition and hormonal fluctuations and menopause. In this study, females placed more importance on nutrition than males (85% “strongly agree” or “agree” that a training specific nutrition is important for their weightlifting training, compared to 74% of males). Nutrition coaches are being consulted by 26% of the females. Males aged 60 or older had the least awareness of nutritional concerns for exercise (68%), compared to females of similar age (83%). Further research is needed on nutrition practices for the aging athlete.

Strengths and limitations: a strength of this study is that the large population of Masters weightlifters in these countries were sampled and, thus, we were able to include a broader population than studies of participants at international competitions and, thus, have a wide range of ages not limited by the constituent year effect. Countries with large participation rates of both males and females in World Master Weightlifting Championships were chosen for this survey. We believe that by including several countries, we have increased the external validity of the findings. However, respondents from the USA were overrepresented. We dealt with this limitation by reporting training variables by region. Another strength of this study is the comparison of geographic regions because of the historic developments of the sport of weightlifting in different regions. Future research should include all countries of the IWF Masters. There are several limitations. There has been attrition of Master athletes in national sports organizations because of the COVID-19 pandemic and there could be a selection bias since the data collection took place more than 1 year since the start of the pandemic. Demographically, the USA participants in June 2021 were slightly older compared to in January 2020 [2], but the balance between females and males remained the same. The questions regarding training and sport participation referred to typical training weeks pre-pandemic, but we do not know to what degree the responses were influenced by the changes brought on by the pandemic. The self-reported frequency and length of weightlifting training sessions were similar in the two years. A comparison is included in Appendix A Table A1. Methods of dissemination of the survey via email and social media differed between countries but reflected typical formats of communication between the national organizations and their members. This was an anonymous online survey and more detailed information on training programs, such as training load, intensity, periodization, recovery, or performance, were not available. Thus, recommendations for optimal training regimens cannot be made. Future studies are needed to address such aspects in competitive Masters weightlifters.

## 5. Conclusions

The weightlifting training of Master athletes is remarkably consistent for males and females and across all ages. Masters weightlifters typically train 3 to 4 days per week, but 5 days is also common for 21% of the participants. After age 50 years, females continue training on average 4 days per week, while the training frequency for males decreases to 3 days per week. A typical training session lasts 1.5 to 2 h for males and females across all ages. This amounts to about 6 to 10 h of weightlifting training per week. Most athletes add other physical activities to their weightlifting training programs. Many females were active in CrossFit before starting weightlifting training and continue to participate in that sport in addition to weightlifting training. Most of the Masters athletes are aware of the importance of nutrition to support optimal weightlifting performance but there seems to be less awareness in older men. European weightlifters deviate in training regimens and attitudes toward nutrition from weightlifters in other regions.

Coaches, athletes, and medical professionals should be aware of the large variation in training practices among older weightlifters. Coaching Masters athletes requires different approaches than coaching younger weightlifters and there may be sex differences [17]. Muscle physiology changes in the aging athlete and exercise regimens and nutrition are modifiable contributing factors [51]. Some practical aspects to consider for the training of older weightlifters are:

- In our study we found that older female weightlifters may have capacity for a higher training frequency than older males,
- Concurrent aerobic physical activities could be added to the training program to reach WHO guidelines to help maintain health and fitness at older ages without it being detrimental to the strength gains [35],
- Engagement in concurrent training and other sports needs to be considered to ensure proper recovery and avoid overtraining,
- More emphasis could be put on nutrition. Most females in this study were aware of its benefits on weightlifting training and recovery, but that awareness was lacking in some older male weightlifters.

Further research with more detailed training diaries and studying markers of cardiovascular health is needed for competitive Masters weightlifters to develop guidance for optimal training programs and to understand health-related outcomes.

## Figures and Tables

**Table 1:** Demographics by age and sex.

	Age 35–44 (*n* = 399)		Age 45–59 (*n* = 422)		Age 60+ (*n* = 230)	
	Females (*n* = 228)	Males (*n* = 171)	Females (*n* = 222)	Males (*n* = 200)	Females (*n* = 73)	Males (*n* = 157)
Region						
Australia	8.8% (20)	9.4% (16)	8.1% (18)	7.0% (14)	11.0% (8)	8.9% (14)
Canada	14.5% (33)	7.0% (12)	11.7% (26)	11.0% (22)	13.7% (10)	13.4% (21)
Europe	11.4% (26)	21.1% (36)	12.2% (27)	30.0% (60)	6.8% (5)	21.0% (33)
USA	65.4% (149)	62.6% (107)	68.0% (151)	52.0% (104)	68.5% (50)	56.7% (89)
Education level						
low	4.4% (10)	8.2% (14)	7.7% (17)	12.6% (25)	12.5% (9)	10.3% (16)
middle	14.0% (32)	18.1% (31)	14.5% (32)	18.6% (37)	25.0% (18)	23.1% (36)
high	47.4% (108)	39.8% (68)	38.5% (85)	32.2% (64)	27.8% (20)	28.2% (44)
graduate degree	34.2% (78)	33.9% (58)	39.4% (87)	36.7% (73)	34.7% (25)	38.5% (60)
Age, Start weightlifting ^1^	34 (30, 36)	32 (28, 36)	44 (40, 48)	37 (15, 45)	55 (49, 60)	19 (14, 55)
Participation in competitions	93.4% (213)	94.7% (162)	92.8% (206)	90.5% (181)	91.8% (67)	89.8% (141)
Referee	19.3% (44)	22.8% (39)	25.7% (57)	27.5% (55)	30.1% (22)	40.1% (63)
Coach	21.1% (48)	25.7% (44)	21.2% (47)	30.0% (60)	15.1% (11)	26.1% (41)
Distance from training location						
home	13.2% (30)	16.4% (28)	14.9% (33)	19.0% (38)	11.0% (8)	29.3% (46)
<30 min	69.7% (159)	69.6% (119)	73.3% (162)	70.0% (140)	74.0% (54)	63.7% (100)
30–60 min	15.4% (35)	12.9% (22)	10.9% (24)	10.5% (21)	12.3% (9)	5.1% (8)
1–1.5 h	1.3% (3)	0.6% (1)	0.9% (2)	0.5% (1)	2.7% (2)	1.3% (2)
>1.5 h	0.4% (1)	0.6% (1)	0.0% (0)	0.0% (0)	0.0% (0)	0.6% (1)

^1^ median (1st, 3rd quartile).

**Table 2:** Training variables by age and sex.

	Age 35–44 (*n* = 399)		Age 45–59 (*n* = 422)		Age 60+ (*n* = 230)	
	Females (*n* = 228)	Males (*n* = 171)	Females (*n* = 222)	Males (*n* = 200)	Females (*n* = 73)	Males (*n* = 157)
Training location ^1^						
Weightlifting Club	58% (132)	64% (108)	56% (124)	56% (111)	63% (46)	54% (83)
CrossFit Box	55% (124)	51% (86)	50% (112)	36% (72)	40% (29)	18% (28)
Fitness Center	15% (33)	21% (36)	15% (34)	15% (29)	19% (14)	20% (31)
Home	21% (48)	36% (60)	21% (48)	36% (71)	22% (16)	40% (61)
Training program ^2^						
coach	68% (156)	52% (89)	70% (155)	45% (90)	76% (55)	33% (51)
remote coach	23% (52)	22% (38)	22% (48)	22% (43)	12% (9)	3% (5)
own	11% (24)	42% (71)	12% (27)	46% (91)	18% (13)	68% (105)
Training days						
1-2 days	7% (17)	7% (12)	8% (19)	15% (29)	9% (7)	17% (25)
3 days	22% (51)	33% (56)	27% (59)	36% (72)	37% (27)	55% (84)
4 days	32% (73)	28% (48)	35% (77)	24% (48)	29% (21)	22% (34)
5 days	32% (72)	24% (41)	24% (54)	19% (38)	19% (14)	5% (7)
6–7 days	6% (15)	8% (13)	6% (13)	7% (1)	5% (4)	3% (4)
Training time						
<1 h	7% (15)	4% (6)	3% (7)	5% (10)	10% (7)	10% (15)
1–1.5 h	26% (60)	30% (50)	35% (77)	30% (60)	37% (27)	39% (59)
1.5–2 h	49% (112)	43% (73)	43% (95)	47% (93)	37% (27)	43% (66)
>2 h	18% (41)	24% (40)	19% (42)	18% (35)	16% (12)	8% (13)
Training time, hours/week ^3^	8 (6, 10)	7.5 (6, 10)	7.5 (6, 10)	6 (5, 8)	6 (4.5, 8)	6 (4.5, 6)
Warm-up						
0–15 min	63% (143)	58% (98)	60% (134)	56% (111)	63% (46)	68% (104)
15–30 min	35% (79)	40% (68)	34% (76)	37% (74)	34% (25)	27% (41)
>30 min	3% (6)	2% (4)	5% (12)	7% (14)	3% (2)	6% (9)
Competition lifts						
0–15 min	4% (10)	1% (1)	1% (2)	4% (7)	5% (4)	5% (7)
15–30 min	12% (28)	6% (11)	9% (19)	10% (19)	12% (9)	10% (16)
30–45 min	26% (59)	36% (61)	26% (57)	29% (57)	27% (20)	31% (48)
45–60 min	33% (76)	34% (57)	37% (83)	33% (66)	27% (20)	36% (55)
>60 min	24% (55)	24% (40)	27% (61)	25% (49)	27% (20)	18% (28)
Strength exercises						
0–15 min	5% (11)	2% (4)	7% (15)	8% (15)	7% (5)	11% (17)
15–30 min	36% (82)	36% (62)	43% (95)	40% (80)	42% (30)	34% (52)
30–45 min	35% (80)	32% (55)	38% (85)	32% (64)	32% (23)	38% (58)
45–60 min	16% (36)	21% (36)	11% (24)	15% (29)	17% (12)	14% (22)
>60 min	8% (19)	8% (13)	1% (3)	6% (11)	3% (2)	3% (5)
Additional exercises						
0–15 min	39% (90)	44% (75)	50% (111)	53% (104)	47% (34)	63% (96)
15–30 min	46% (106)	38% (65)	40% (88)	37% (74)	40% (29)	29% (45)
30–45 min	10% (22)	12% (20)	7% (16)	8% (16)	11% (8)	7% (10)
45–60 min	4% (8)	5% (8)	1% (2)	2% (3)	1% (1)	0% (0)
>60 min	1% (2)	1% (2)	2% (4)	1% (1)	0% (0)	1% (2)
Cool-down						
0–15 min	93% (211)	91% (153)	94% (208)	90% (180)	93% (67)	91% (135)
15–30 min	7% (16)	8% (13)	6% (13)	8% (15)	6% (4)	9% (13)
>30 min	0% (0)	2% (3)	0% (1)	2% (4)	1% (1)	1% (1)
Nutrition						
Strongly agree	54% (123)	46% (78)	50% (112)	43% (85)	53% (39)	38% (57)
Agree	32% (72)	34% (58)	35% (78)	33% (66)	30% (22)	30% (45)
Neither agree nor disagree	11% (25)	15% (26)	12% (26)	18% (36)	15% (11)	25% (38)
Disagree	3% (6)	4% (6)	2% (5)	4% (8)	1% (1)	7% (10)
Strongly disagree	1% (2)	1% (1)	0% (1)	2% (4)	0% (0)	1% (2)
Nutrition program ^2^						
coach	27.6% (63)	10.1% (17)	25.7% 57)	12.6% (25)	21.4% (15)	4.5% (7)
own	29.8% (68)	42.0% (71)	29.7% (66)	37.2% (74)	37.1% (26)	40.3% (62)
advice	13.2% (30)	26.6% (45)	16.2% (36)	24.6% (49)	24.3% (17)	22.7% (35)

^1^ multiple locations possible; ^2^ combination of programs possible; ^3^ median (1st, 3rd quartile).

**Table 3:** Training variables by region.

	AUS (*n* = 90)		CAN (*n* = 124)		USA (*n* = 650)		EUR (*n* = 187)	
	Females (*n* = 46)	Males (*n* = 44)	Females (*n* = 69)	Males (*n* = 55)	Females (*n* = 350)	Males (*n* = 300)	Females (*n* = 58)	Males (*n* = 129)
Training location ^1^								
Weightlifting Club	58.7% (27)	45.5% (20)	66.7% (46)	71.7% (38)	53.0% (185)	48.1% (143)	75.9% (44)	80.2% (101)
CrossFit Box	45.7% (21)	36.4% (16)	47.8% (33)	24.5% (13)	53.3% (186)	45.5% (135)	43.1% (25)	17.5% (22)
Fitness Center	15.2% (7)	22.7% (10)	17.4% (12)	15.1% (8)	15.2% (53)	21.9% (65)	15.5% (9)	10.3% (13)
Home	34.8% (16)	45.5% (20)	17.4% (12)	47.2% (25)	22.1% (77)	41.8% (124)	10.3% (6)	17.5% (22)
Training program ^2^								
coach	87.0% (40)	50.0% (22)	71.0% (49)	48.1% (26)	68.6% (240)	44.5% (133)	63.8% (37)	38.9% (49)
remote coach	15.2% (7)	13.6% (6)	15.9% (11)	18.5% (10)	23.5% (82)	21.7% (65)	15.5% (9)	4.0% (5)
own	4.3% (2)	38.6% (17)	13.0% (9)	46.3% (25)	10.0% (35)	45.2% (135)	31.0% (18)	71.4% (90)
Training days								
1–2 days	13.0% (6)	11.3% (5)	7.2% (5)	14.8% (8)	7.2% (25)	7.0% (21)	12.1% (7)	25.4% (32)
3 days	21.7% (10)	22.7% (10)	34.8% (24)	46.3% (25)	23.4% (82)	37.5% (112)	36.2% (21)	51.6% (65)
4 days	28.3% (13)	34.1% (15)	26.1% (18)	24.1% (13)	34.6% (121)	27.4% (82)	32.8% (19)	15.9% (20)
5 days	23.9% (11)	25.0% (11)	26.1% (18)	11.1% (6)	29.4% (103)	20.4% (61)	13.8% (8)	6.3% (8)
6–7 days	13.0% (6)	6.8% (3)	5.7% (4)	3.8% (2)	5.4% (19)	7.7% (23)	5.2% (3)	0.8% (1)
Training time								
<1 h	4.3% (2)	11.4% (5)	1.4% (1)	3.8% (2)	7.1% (25)	5.7% (17)	1.8% (1)	5.6% (7)
1–1.5 h	43.5% (20)	40.9% (18)	37.7% (26)	24.5% (13)	29.7% (104)	36.2% (108)	24.6% (14)	24.0% (30)
1.5–2 h	37.0% (17)	36.4% (16)	44.9% (31)	60.4% (32)	43.4% (152)	44.0% (131)	59.6% (34)	42.4% (53)
>2 h	15.2% (7)	11.4% (5)	15.9% (11)	11.3% (6)	19.7% (69)	14.1% (42)	14.0% (8)	28.0% (35)
Training time, hours/week ^3^	7.5 (6, 9)	7.5 (4.5, 8)	7.5 (6, 8)	6 (4.5, 8)	8 (6, 10)	6 (6, 9)	6 (6, 8)	6 (4, 7.5)
Warm-up								
0–15 min	71.7% (33)	63.6% (28)	63.8% (44)	55.6% (30)	58.3% (204)	55.9% (167)	72.4% (42)	69.8% (88)
15–30 min	28.3% (13)	31.8% (14)	30.4% (21)	37.0% (20)	37.4% (131)	39.1% (117)	25.9% (15)	25.4% (32)
>30 min	0.0% (0)	4.5% (2)	5.8% (4)	7.4% (4)	4.3% (15)	5.0% (15)	1.9% (1)	4.8% (6)
Competition lifts								
0–15 min	4.3% (2)	4.5% (2)	2.9% (2)	3.7% (2)	3.4% (12)	3.0% (9)	0.0% (0)	1.6% (2)
15–30 min	17.4% (8)	11.4% (5)	8.7% (6)	5.6% (3)	9.4% (33)	9.1% (27)	15.5% (9)	8.7% (11)
30–45 min	23.9% (11)	29.5% (13)	30.4% (21)	31.5% (17)	23.7% (83)	31.2% (93)	36.2% (21)	34.1% (43)
45–60 min	30.4% (14)	34.1% (15)	37.7% (26)	35.5% (19)	34.3% (120)	36.2% (108)	32.8% (19)	28.6% (36)
>60 min	23.9% (11)	20.5% (9)	20.3% (14)	24.1% (13)	29.1% (102)	20.5% (61)	15.5% (9)	27.0% (34)
Strength exercises								
0–15 min	0.0% (0)	13.6% (6)	5.9% (4)	3.7% (2)	5.4% (19)	5.7% (17)	13.8% (8)	8.7% (11)
15–30 min	50.0% (23)	40.9% (18)	42.6% (29)	40.7% (22)	38.6% (135)	35.8% (107)	34.5% (20)	37.3% (47)
30–45 min	21.7% (10)	25.0% (11)	29.4% (20)	31.5% (17)	39.4% (138)	33.8% (101)	34.5% (20)	38.1% (48)
45–60 min	28.3% (13)	15.9% (7)	16.2% (11)	20.4% (11)	11.7% (41)	17.7% (53)	12.1% (7)	12.7% (16)
>60 min	0.0% (0)	4.5% (2)	5.9% (4)	3.7% (2)	4.9% (17)	7.0% (21)	5.2% (3)	3.2% (4)
Additional exercises								
0–15 min	47.8% (22)	61.4% (27)	47.1% (32)	53.7% (29)	44.9% (157)	52.3% (156)	42.1% (24)	50.4% (63)
15–30 min	45.7% (22)	34.1% (15)	35.3% (24)	32.0% (20)	44.3% (155)	36.6% (109)	40.4% (23)	36.6% (40)
30–45 min	4.3% (2)	2.3% (1)	14.7% (10)	7.4% (4)	8.6% (30)	8.1% (24)	7.0% (4)	8.1% (17)
45–60 min	2.2% (1)	0.0% (0)	0.0% (0)	0.0% (0)	2.0% (7)	2.0% (6)	5.3% (3)	4.0% (5)
>60 min	0.0% (0)	2.3% (1)	2.9% (2)	1.9% (1)	0.3% (1)	1.0% (3)	5.3% (3)	0.0% (0)
Cool-down								
0–15 min	97.8% (44)	90.9% (40)	97.1% (66)	90.7% (49)	92.3% (323)	90.6% (269)	91.4% (53)	90.2% (110)
15–30 min	2.2% (1)	9.1% (4)	2.9% (2)	7.4% (4)	7.4% (26)	8.1% (24)	6.9% (4)	7.4% (9)
>30 min	0.0% (0)	0.0% (0)	0.0% (0)	1.9% (1)	0.3% (1)	1.3% (4)	1.7% (1)	2.5% (3)
Nutrition								
Strongly agree	52.2% (24)	47.7% (21)	60.9% (42)	40.7% (22)	54.0% (189)	49.7% (147)	32.8% (19)	23.8% (30)
Agree	34.8% (16)	38.6% (17)	27.5% (19)	24.1% (13)	31.7% (111)	34.1% (102)	44.8% (26)	30.2% (38)
Neither agree nor disagree	8.7% (4)	13.6% (6)	10.1% (7)	29.6% (16)	12.0% (42)	12.8% (38)	15.5% (9)	31.7% (40)
Disagree	4.3% (2)	0.0% (0)	1.4% (1)	3.7% (2)	1.7% (6)	2.7% (8)	5.2% (3)	11.1% (14)
Strongly disagree	0.0% (0)	0.0% (0)	0.0% (0)	1.9% (1)	0.6% (2)	0.7% (2)	1.7% (1)	3.2% (4)
Nutrition program ^2^								
coach	26.1% (12)	9.1% (4)	28.4% (19)	7.4% (4)	26.9% (94)	11.7% (35)	17.2% (10)	4.8% (6)
own	19.6% (9)	40.9% (18)	31.3% (21)	40.7% (22)	31.8% (111)	43.8% (131)	32.8% (19)	28.8% (36)
advice	19.6% (9)	29.5% (13)	13.4% (9)	25.9% (14)	15.5% (54)	20.4% (61)	19.0% (11)	32.8% (41)
Cross-training								
None	34.8% (16)	29.5% (13)	24.6% (17)	30.9% (17)	29.1% (102)	33.7% (101)	38.6% (22)	46.8% (59)
CrossFit	41.3% (19)	31.8% (14)	49.3% (34)	29.1% (16)	46.9% (164)	32.7% (98)	31.0% (18)	15.5% (20)
Endurance (running, swimming, cycling, walking)	19.6% (9)	20.5% (9)	21.7% (15)	29.1% (16)	25.4% (89)	25.7% (77)	19.0% (11)	30.2% (39)
Fitness	28.3% (13)	25.0% (11)	23.2% (16)	30.9% (17)	20.0% (70)	21.3% (64)	15.5% (9)	13.2% (17)
Ball sports	6.5% (3)	11.4% (5)	5.8% (34	20.0% (11)	5.4% (19)	14.0% (42)	1.7% (1)	9.3% (12)
Yoga/Pilates	8.7% (4)	2.3% (1)	20.3% (14)	7.3% (4)	22.0% (77)	6.0% (18)	12.1% (7)	1.6% (2)

^1^ multiple locations are possible; ^2^ a combination of programs is possible; ^3^ estimated from days and maximum session length.

**Table 4:** Sports prior to weightlifting by age and sex.

	Age 35–44 (*n* = 399)		Age 45–59 (*n* = 422)		Age 60+ (*n* = 230)	
	Females (*n* = 228)	Males (*n* = 171)	Females (*n* = 222)	Males (*n* = 200)	Females (*n* = 73)	Males (*n* = 157)
No prior sport	5.3% (12)	6.4% (11)	9.5% (21)	9.0% (18)	15.1% (11)	7.1% (11)
Strength training	39.9% (91)	32.2% (55)	39.6% (88)	41.0% (82)	26.0% (19)	43.9% (69)
Power lifting	11.4% (26)	22.2% (38)	15.3% (34)	17.5% (35)	11.0% (8)	20.4% (32)
CrossFit	75.0% (171)	63.2% (108)	69.8% (155)	42.5% (85)	41.1% (30)	24.8% (39)
Ball sports	35.1% (80)	49.7% (85)	29.3% (65)	53.5% (107)	13.7% (10)	44.6% (70)
Track and field	17.1% (39)	24.6% (42)	15.3% (34)	18.5% (37)	13.7% (10)	32.5% (51)
Endurance (running, swimming, cycling, walking)	41.2% (94)	37.4% (64)	49.5% (110)	31.0% (62)	41.4% (30)	24.8% (39)
Martial arts, wrestling, boxing	10.5% (24)	22.2% (38)	12.6% (28)	15.5% (31)	2.7% (2)	13.4% (21)
Fitness	37.3% (85)	31.6% (54)	40.5% (90)	30.5% (61)	39.7% (29)	17.8% (28)
Gymnastics	9.6% (22)	0.0% (0)	6.8% (15)	1.0% (2)	1.4% (1)	4.5% (7)
Yoga/Pilates	22.4% (51)	5.8% (10)	25.2% (56)	6.5% (13)	20.5% (15)	2.5% (4)
Other	15.4% (35)	9.9% (17)	13.5% (30)	13.0% (26)	16.4% (12)	17.8% (28)

**Table 5:** Sports in addition to weightlifting by age and sex.

	Age 35–44 (*n* = 399)		Age 45–59 (*n* = 422)		Age 60+ (*n* = 230)	
	Females (*n* = 228)	Males (*n* = 171)	Females (*n* = 222)	Males (*n* = 200)	Females (*n* = 73)	Males (*n* = 157)
No additional sport	32.5% (74)	35.1% (60)	29.0% (64)	32.5% (65)	26.0% (19)	42.2% (65)
Strength training	10.1% (23)	14.0% (24)	10.4% (23)	17.0% (34)	15.1% (11)	14.6% (23)
Power lifting	4.4% (10)	9.4% (16)	8.1% (18)	5.5% (11)	4.1% (3)	5.1% (8)
CrossFit	47.8% (109)	18.6% (66)	46.4% (103)	30.5% (61)	31.5% (23)	13.4% (21)
Ball sports	7.9% (18)	15.2% (26)	3.6% (8)	15.0% (30)	1.4% (1)	8.9% (14)
Track and field	1.3% (3)	4.1% (7)	0.9% (2)	3.0% (6)	2.7% (2)	5.7% (9)
Endurance (running, swimming, cycling, walking)	21.5% (49)	26.9% (46)	23.9% (53)	28.0% (56)	30.1% (22)	24.8% (39)
Martial arts, wrestling, boxing	2.6% (6)	5.8% (10)	1.8% (4)	3.5% (7)	0.0% (0)	2.5% (4)
Fitness	16.7% (38)	21.1% (36)	25.2% (56)	24.0% (48)	19.2% (14)	15.9% (25)
Yoga/Pilates	18.9% (43)	5.8% (10)	20.7% (46)	6.0% (12)	17.8% (13)	1.9% (3)
Other	3.9% (9)	4.1% (7)	4.1% (9)	5.0% (10)	5.5% (4)	7.6% (12)

## Data Availability

All relevant data are within the manuscript.

## Appendix A

The USA surveys were conducted in January 2020 (pre-pandemic [2]) and in June 2021, after the COVID-19 pandemic was already in place for over a year. Survey questions in 2021 referred to typical training weeks pre-pandemic, so responses relied on recall.

The participants in 2021 were slightly older (mean 50.2 versus 47.5 years in 2020, *p* < 0.001), but had similar proportions of females and males. In both years the athletes primarily followed a program by a coach, but a larger proportion in 2021 also used their own program to supplement or on its own (26.2% vs. 17.7%, *p* < 0.001). However, the frequency and length of training sessions were similar in both years. In 2021, a larger proportion of athletes reported that they did not participate in physical activities in addition to weightlifting (33.7%) compared to 2020 (18.8%), but the participation in CrossFit was similar (40.3% versus 39.3%, *p* = 0.687).

**Table A1:** Comparison of USA respondents to surveys conducted in January 2020 and in June 2021.

	USA 2020 (*n* = 949)	USA 2021 (*n* = 650)	*p*-Value
Age, mean ± sd	47.5 ± 10.3	50.2 ± 11.3	<0.001
Age groups, % (*n*)			<0.001
35–44	47.4% (450)	39.4% (256)	
45–59	38.3% (363)	39.2% (255)	
60+	14.3% (136)	21.4% (139)	
Sex, male % (*n*)	45.3% (429)	46.2% (300)	0.737
Education, *n* (%)			<0.001
low	0.1% (1)	6.0% (39)	
middle	17.8% (169)	9.4% (61)	
high	39.7% (377)	35.8% (232)	
graduate school	42.4% (402)	48.8% (316)	
Training program ^1^			
coach (in-person or remote)	75.9% (720)	75.8% (491)	1.0
own	17.7% (168)	26.2% (170)	<0.001
Training location ^2^			
Weightlifting Club	47.0% (446)	50.8% (328)	0.138
CrossFit Box	41.6% (395)	49.7% (321)	0.001
Fitness Center	13.4% (127)	18.3% (118)	0.008
Home	29.9% (284)	31.1% (201)	0.613
Training days			0.220
1–2 days	5.7% (59)	7.1% (46)	
3 days	25.6% (243)	29.9% (194)	
4 days	33.9% (322)	31.3% (203)	
5 days	27.5% (261)	25.3% (164)	
6–7 days	6.8% (64)	6.5% (42)	
Training time			0.213
<1 h	12.2% (116)	6.5% (42)	
1–1.5 h	34.0% (322)	32.7% (212)	
1.5–2 h	37.5% (355)	43.7% (283)	
>2 h	16.3% (154)	17.1% (111)	
Training time hours/week ^3^	7.5 (5,10)	7.5 (6,10)	0.345
Cross-training			
None	18.7% (177)	33.7% (219)	<0.001
CrossFit	39.3% (373)	40.3% (262)	0.687
Endurance (running, swimming, cycling, walking)	41.4% (392)	25.5% (166)	<0.001
Ball sports	7.5% (71)	9.4% (61)	0.174
Yoga/Pilates	19.8% (188)	14.6% (95)	0.008

^1^ a combination of programs is possible; ^2^ multiple locations are possible; ^3^ estimated from days and maximum session length.
